# Electromechanical Characteristics by a Vertical Flip of C_70_ Fullerene Prolate Spheroid in a Single-Electron Transistor: Hybrid Density Functional Methods

**DOI:** 10.3390/nano11112995

**Published:** 2021-11-08

**Authors:** Jong Woan Choi, Changhoon Lee, Eiji Osawa, Ji Young Lee, Jung Chul Sur, Kee Hag Lee

**Affiliations:** 1 Department of Semiconductor and Display, Wonkwang University, Iksan 54538, Korea; jangja21@wku.ac.kr; 2 Max Planck POSTECH Center for Complex Phase of Materials, Pohang University of Science and Technology, Pohang 37673, Korea; 3 NanoCarbon Research Institute, AREC, Shinshu University, Ueda, Nagano 386-8567, Japan; osawa@nano-carbon.jp; 4 Department of Chemistry, Nanoscale Sciences and Technology Institute, Nanocarbon R&D Institute, Wonkwang University, Iksan 54538, Korea

**Keywords:** hybrid density functional theory, electromechanical characteristics, C_70_ fullerene, point charges model, C_70_ vertical flip isomers facing the gate electrode between the electrodes

## Abstract

In this study, the B3LYP hybrid density functional theory was used to investigate the electromechanical characteristics of C_70_ fullerene with and without point charges to model the effect of the surface of the gate electrode in a C_70_ single-electron transistor (SET). To understand electron tunneling through C_70_ fullerene species in a single-C_70_ transistor, descriptors of geometrical atomic structures and frontier molecular orbitals were analyzed. The findings regarding the node planes of the lowest unoccupied molecular orbitals (LUMOs) of C_70_ and both the highest occupied molecular orbitals (HOMOs) and the LUMO of the C_70_ anion suggest that electron tunneling of pristine C_70_ prolate spheroidal fullerene could be better in the major axis orientation when facing the gate electrode than in the major (longer) axis orientation when facing the Au source and drain electrodes. In addition, we explored the effect on the geometrical atomic structure of C_70_ by a single-electron addition, in which the maximum change for the distance between two carbon sites of C_70_ is 0.02 Å.

## 1. Introduction

Using molecules as electronic components in single-molecule transistors (SMTs) is a powerful new direction in the field of nanometer-scale systems [1,2]. The emerging field of molecular electronics (ME) based on single molecules offers a platform for the further miniaturization of devices that can respond to various external excitations. Thus, molecular electronic systems are ideal for the study of charge transport on a single-molecule scale. The design of functional molecular devices has moved the study of metal–molecule–metal junctions beyond classic electronic transport characterization. Changes in the conductance of single-molecule junctions in response to various external stimuli is crucial regarding the study of single-molecule electronic devices that can be combined to provide multiple functionalities [3,4,5].

Studying transport through a single molecule in a single-electron transistor (SET) involves probing molecules in a two-terminal geometry using mechanically controlled break junctions [6] or scanning probes [7], such as for C_60_ molecules [8,9]. Transistors containing a transition metal complex were designed in a previous study to undergo electron transport via well-defined states of charge of a single atom and examined with two related molecules containing a Co ion bonded to polypyridyl ligands attached to insulating tethers of different lengths [10]. Here, changing the length of the insulating tether changes the coupling between the ion and the electrodes, implying that it would be possible to fabricate devices that exhibit single-electron phenomena.

To date, fullerenes have attracted much attention because they are thought to be good candidates for the construction of highly conductive molecular junctions since they were first evaluated using a scanning tunneling microscope [7]. It is well known that the electrical conductance of the system does not change unless there are variations in its shape, size, and composition due to some external stimulus. The number of contact points between the device electrodes and the molecule plays an important role in electron conduction. Resonance tunneling occurs when an electrode contacts a carbon atom in a molecule, implying that the gate voltage can shift the molecular levels of a C_60_ fullerene molecule to control the transmission [11]. Fullerenes have many potential applications in molecular electronic devices, witnessed by the first single-molecule electromechanical amplifier [12], single-molecule transistor [8], and a molecular data storage system [13]. In addition, the transport of C_60_-based junctions can be adjusted through doping [14].

The first demonstration of a molecular SET was a device consisting of C_60_ coupled to gold electrodes fabricated by electromigration [8]. The gate electrodes were oxidized degenerately doped silicon substrates. This study presented evidence of transport through a single molecule by a Coulomb blockade with a gap of 150 meV. In addition, two vibration modes were observed measuring 33 meV, resulting from the internal vibration of C_60_, and 5 meV, corresponding to the motion of the C_60_ molecule in the van der Waals potential of the gold electrodes. Small (1 meV) energy division is observed in many lines. This splitting may occur from the C_60_ center-of-mass motion perpendicular to the surface normal discussed above. Unfortunately, the potential characteristics of this motion are unknown due to the lack of detailed knowledge of the shape of electrodes near C_60_ and thus the lack of quantitative support for this assignment.

In addition, using the electromigration technique, evaluations of single C_70_ and C_140_ molecules showed that an excited level near 3 meV of a C_70_ device is attributed to the ‘bouncing-ball’ mode of C_70_ on a gold surface, as previously observed in C_60_ transistors [8,15], and that an 11 meV level observed in C_140_ devices is intrinsic to C_140_, because no energy levels near 11 meV were observed in C_70_ devices [15]. The average energy of the bouncing-ball mode from several C_70_ devices was approximately 4 meV, which is consistent with a simple harmonic potential approximation for the van der Waals interaction between a C_70_ molecule and a gold surface, whereas the energy of 11 meV was considered to be the intercage vibrational stretching mode of the C_70_ dimer. In a different study, a histogram of all of the excited state energies below 20 meV indicated that an excitation at 11 ± 1 meV was observed in 11 of 14 C_140_ devices but in only 1 of 8 C_70_ devices [16], implying that 78.6% of C_140_ and 12.5% of C_70_ devices showed an energy mode of 11 meV. The orientation of the molecule in the device was not specified. All measurements were performed either at temperatures of 1.5 K or below 0.1 K.

Venkataraman et al. demonstrated that molecules such as bipyridine can assemble under different geometries in a metal junction, which affects the junction conductance [17]. The conductance histograms of bipyridine-based molecular wires exhibit double-peak characteristics owing to their two different binding geometries. This allows mechanical switching between two defined conductance states by changing the distance in a mechanically controlled break junction. Using scanning tunneling microscopy, the vibronic states of single C_60_ and C_70_ molecules were observed [18,19]. It was concluded that the vibronic progressions were sensitive to the molecular orientations and can have different intervals in different electronic bands of the same molecule. 

To the best of our knowledge, the energy mode of 11 meV that was reported in a single C_70_ molecule transistor device in [16] has not been studied in detail to date. Thus, it should be interesting to understand the mechanical switching based on the energy mode of 11 meV involving the conductance between two defined states. Therefore, we explore the coupling mode of conductance between C_70_ fullerene and gold electrodes by changing the orientation through a vertical flip from the major (longer) axis to the minor axis of C_70_ fullerene in this study. We evaluate C_70_ vertical flip isomers facing the gate electrode located between two electrodes in a mechanically controlled break junction, implying that an input voltage could be applied to match the energy scale of a vibrationally or rotationally excited molecular level.

Therefore, our goal is to explore the orientation of C_70_ vertical flip isomers facing the gate electrode between two electrodes to explain the 11 meV energy mode of a C_70_ SET using density functional theory methods. Here, the atomic structure of C_70_ fullerene is a prolate spheroid, implying that if the ellipse is rotated about its major (longer) axis, the result is a *prolate* (elongated) spheroid shaped like an American football or a rugby ball. Thus, the biaxial orientation of the C_70_ vertical flip isomers would be two stable isomers from an isomer chemical species viewpoint. We then study the effect of two orientations of C_70_ vertical flip isomers when facing the gate electrode between two electrodes by using the hybrid density functional theory method and a point charge model. Here, we limit the discussion to molecular wires, in which charge transfer occurs through the p-conjugated part of the molecule, such that electrons can move freely in the delocalized orbitals over the fullerene. The bistability of conductance for a molecular switch generally requires molecules with two stable isomers, and the transition between isomers is controlled by external stimuli (e.g., light, heat, current, or an electric field).

## 2. Materials and Methods

Hybrid Density Functional Calculations

In this study, hybrid density functional theory, using Becke’s three-parameter hybrid method combined with the Lee–Yang–Parr exchange-correlation functional theory (B3LYP) [20,21], was applied to fully optimize the geometries of C_70_ and its anion, without constraints, by applying the convergence criterion with tight optimization and an ultrafine pruned grid (99,590 elements) at the level of the electron basis set 6–31G (d, p) [22] with and without GD3 [23]. Here, GD3 is the D3 version of Grimme’s dispersion with the original D3 damping function.

Physical adsorption by van der Waals forces between the substrate and fullerene would be approximately 10 kcal/mol. Thus, in a single-molecule device, a single molecule could be oriented in a specific direction between the electrodes under an experimental environment at low temperature. The value of the effective van der Waals radius of an atom in a crystal depends on the strength of the attractive forces holding the molecules together and also on the orientation of the contact relative to the covalent bond or bonds between atoms; accordingly, it is much more variable than the corresponding covalent radius. The van der Waals radii can be taken as equal to the single-bond covalent radius plus 0.80 Å as their limit of reliability [24].

Here, we have conducted all the calculations by using the Gaussian09 D.01 package suite [25]. Then, we calculated the energies for the skeletal rearrangements of the orientation of C_70_ vertical flip isomers facing the gate electrode between two electrodes, consisting of the C_70_ fullerene system and the point charges as a model for the surface [26,27] of the gate electrode in a SET through single-point calculations involving the geometries of C_70_ and its anion by applying the convergence criterion with tight optimization.

Here, the B3LYP/6-31G* level by including and not including empirical dispersion interaction GD3 has been employed to study C_70_ fullerene systems because it yields reliable geometries and energies [28,29,30]. Partial atomic charges were calculated based on Mulliken’s population analysis [31] of the optimized anion and neutral C_70_. All the optimized structures exhibited real vibrational frequencies, and their Cartesian coordinates are reported in the Supplementary Material (See Table S1). 

## 3. Results and Discussion

### 3.1. Geometries of C_70_ and Its Anion Species

**Figure 1:**
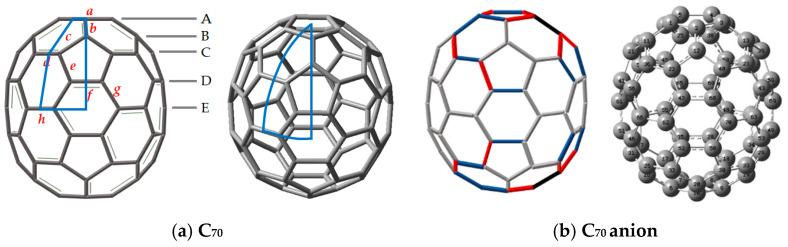
(**a**) The five inequivalent atoms in the D_5h_ structure of the C_70_ neutral fullerene molecule are denoted by lowercase letters. In C_70_, the bond lengths (***a***, ***b***, ***c***, ***d***, ***e***, ***f***, ***g***, and ***h***) are as follows: 1.452, 1.397, 1.449, 1.389, 1.450, 1.434, 1.421, and 1.470 Å, respectively. Here, **A**–**E** levels represent five different types of carbon atoms from a curvature viewpoint. The representative patch (blue line region) in (**a**) is the unit cell of C_70_ with respect to the D_5h_ point group symmetry. (**b**) The point group of C_70_ anion is C_2v_ reduced from D_5h_ symmetry, which is represented with colors on the unit cell.

The optimized bond distances of the geometries of C_70_ and its anion for the ground state C_70_ chemical species, as shown in Figure 1, were obtained using B3 (Becke’s three-parameter formulation hybrid method) and the Lee–Yang–Parr (LYP) exchange-correlation functional theory (B3LYP) [20,21] with the electron basis set 6–31G (d, p) [22] without and with GD3, which is the D3 version of Grimme’s dispersion with the original D3 damping function [23].

The C–C bond C_5_ rotation symmetry operation could support structural identification in the case of symmetrical fullerenes (see Figure 1). The prolate spheroidal C_70_ molecule with D_5h_ symmetry is shown in Figure 1a, in which a representative (green or blue) patch on its surface emphasizes its D_5h_ symmetry. The area of the patch outlined in blue is equal to 1/20 of the total surface area. There are 18 key points on the C_70_ (D_5h_) representative patch: five different types of carbon atoms, eight distinct C–C bonds, three types of six-member rings, and two types of five-member rings.

When 20 elements of the D_5h_ group are included in the patch, the patch encompasses the entire surface of the polyhedron. Thus, we consider the patch on the surface of C_70_ (D_5h_) as the smallest structural unit instead of considering the whole cage. In Figure 1, C_70_ has eight types of the bond lengths, designated as ***a***, ***b***, ***c***, ***d***, ***e***, ***f***, ***g***, and ***h***, which measure 1.452, 1.397, 1.449, 1.389, 1.450, 1.434, 1.421, and 1.470 Å, respectively. As shown in Figure 1, the carbons of C_70_ fall into the five groups labeled from the upper pole (through equatorial regions) to the bottom pole along its major axis, which are named A, B, C, D, and E. Accordingly, we labeled the five stairs as A, B, C, D, and E, representing the five distinct carbon atoms of C_70_ whose curvatures can be different; therefore, each is affected differently by the surface charge of the electrodes (abundances 10:10:20:20:10).

In the case of the C_70_ anion, the carbon–carbon bond lengths are distinguished by the increased, unchanged, and contracted parts of the bond lengths compared to the neutral case on the unit fragment of the C_2v_ symmetry. As shown in Figure 1b, when the bond length is changed by 0.01 Å, we represent it with a single line unit in which the increased length is represented by a blue line, the contracted part by a red line, and the unchanged part by a green line. The 0.02 Å increase of the bond length is indicated by a black line (there are no contractions). Figure 1b indicates that the bond lengths of the equatorial portion experience little effect from single-electron doping (they remain green), while the change in bond lengths around the poles is represented by black, blue, and red lines. Here, colored lines are shown for only one pole in Figure 1b (See Table S1). 

### 3.2. Single-Molecule C_70_ Fullerene-Gate Electrode Interaction in a Single-C_70_ Transistor in Terms of the Point-Charge Approximation

**Figure 2:**
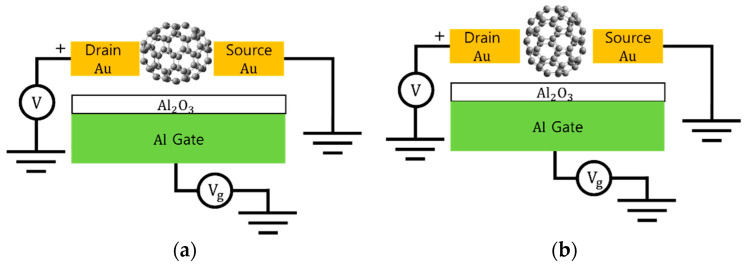
Schematic diagrams of idealized single-C_70_ transistors. The spheroidal geometry of C_70_ fullerene can face the electrodes in two types of major axis and minor axis directions: (**a**) when the minor axial direction of the C_70_ faces the gate electrode surface and (**b**) when the major axial direction faces the gate electrode surface.

As shown in Figure 2, the spheroidal C_70_ fullerene has two specific orientations for the C_70_ vertical flip isomers facing the gate electrode using the prolate spheroid geometry in a conducting field under an external primary electric field, including the surface dielectric polarity charge distribution of the gate electrode. One orientation is its (longer) major axis directed toward the gate electrode, and the other is its major axis directed toward the Au electrodes. A prolate spheroid is a spheroid in which the polar radius (the major axis) is longer than the equatorial radius (the minor axis). Prolate spheroids are similar to symmetrical eggs (i.e., having the same shape at both poles). Thus, two C_70_ vertical flip isomers facing the gate electrode for a C_70_ spheroidal molecule between the electrodes are used to demonstrate whether the energy mode of 11 meV in a single-C_70_ transistor can arise from a change in the orientation of the C_70_ vertical flip isomers facing the gate electrode between the electrodes, implying that the two orientation isomers can be adjusted from the spatial orientation viewpoint to obtain the 11 meV energy mode. Therefore, in order to examine the 11 meV energy mode, we calculated the energy difference between the two orientations of the C_70_ vertical flip isomers facing the gate electrode by using first-principle calculations, DFT simulations, and the point charges to construct a model of the gate electrode surface.

**Figure 3:**
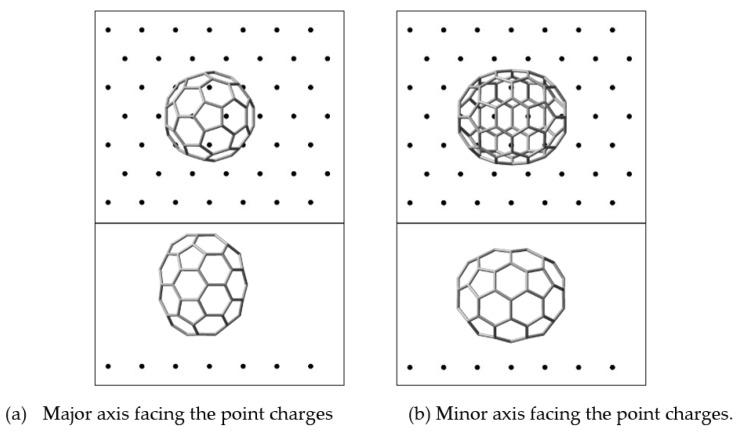
Major axial in (**a**) and minor axial in (**b**) orientation arrangements of a C_70_ fullerene spheroid molecule facing the model surface of the gate electrode made up of point charges located on 7 × 7 mesh points. (**b**) Here, minor axial orientation arrangement of a C_70_ fullerene molecule facing the point charges is a model of the major axis of a C_70_ fullerene spheroid facing the Au source and drain electrodes.

To examine how the stability of the orientation of the C_70_ fullerene between Au electrodes might be affected by the gate electrode, it is necessary to estimate the dependence of the C_70_-gate electrode interaction as a function of both the distance between the fullerene and the gate surface, and the polarity charge of the gate electrode surface induced by the gate voltage. To simulate the effect of the charge of the gate surface, we represent the gate-voltage-induced polarity surface by a single sheet of 49 point charges δ located on a 7 × 7 grid of the Al(111) surface, as shown in Figure 3. Then, we performed first-principle electronic structure calculations with the B3LYP hybrid method including the empirical dispersion interaction GD3 for C_70_ plus the model surface. As shown in Figure 3, we considered two arrangements between C_70_ and the model surface for δ = +0.01 and −0.01. Similar results were obtained for other values examined. Thus, in the following section, we discuss only those based on δ = +0.01 and −0.01.

**Figure 4:**
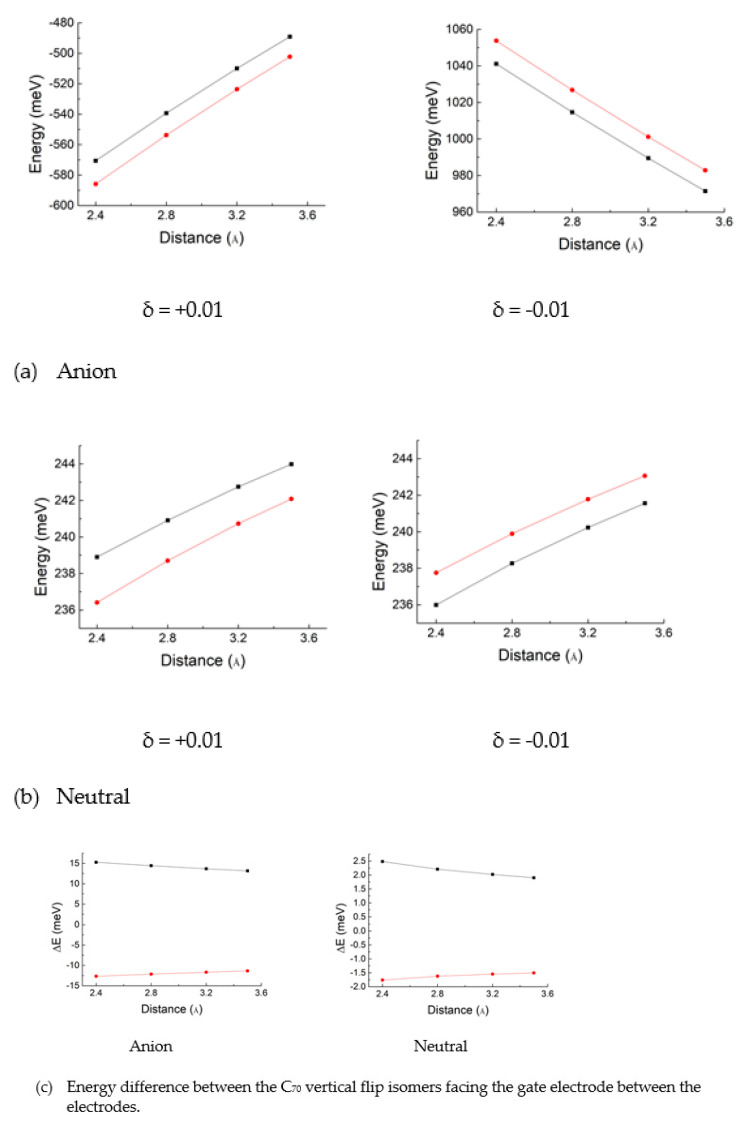
C_70_- point charge interaction energy, ∆*E*, calculated as a function of the distance between the model surface of point charges δ and the closest atoms of C_70_ chemical species to the surface: in (**a**,**b**), a blue line represents the interaction energy between fullerene and a point charge model of the gate electrode, in which the major axis faces the gate electrode surface, and a red line represents when the minor axis faces the gate electrode surface. (**c**) ∆*E* values for δ = −0.01 are represented by filled blue circles, and those for δ = +0.01 by filled red circles. Here, the q values (=−0.01 and 0.01) were chosen to be the energy barrier of approximately 11 meV for the C_70_ vertical flip by simulating q from −0.04 to 0.04 with step 0.01.

For the C_70_ fullerene plus point charge coupling model, the interaction energy Δ*E* was calculated as a function of the distance ***r*** between the C_70_ fullerene and the model surface, where ***r*** is measured from the surface to the closest atoms of the C_70_ fullerene. The results of our calculations are summarized in Figure 4, where ∆*E* refers to the energy of a C_70_ fullerene molecule interacting with the model surface minus the energy of an isolated C_70_ fullerene molecule. The ∆*E* values for all examined cases are meaningful only in their relative values. That is, the more negative ∆*E* is, the more energetically favorable the conditions for adsorption.

When the major axis of a C_70_ fullerene anion between the Au electrodes faces both the gate electrode surface and the Au electrodes, the positive surface charge is more favorable for adsorption than the negative surface charge (Figure 4a). By contrast, with the major axis facing the gate surface in neutral C_70_, the negative surface charge is more favorable for adsorption than the positive surface charge (Figure 4b). In addition, with the major axis facing the Au electrode surface in neutral C_70_, the positive surface charge is more favorable for adsorption than the negative surface charge (Figure 4b), similar to its anion.

As shown in Figure 4c, the energy difference between the major axis of a C_70_ fullerene anion facing the gate electrode surface and the major axis of anion facing the Au electrodes represents that the case of the major axis of anion facing the gate electrode is more favorable for adsorption on the negative surface charge than on the positive surface charge. By contrast, with the major axis facing the Au electrode surface considering the C_70_ anion, the positive surface charge is more favorable for adsorption than the negative surface charge (Figure 4a,c). Moreover, when we compare the case when the major axis faces the gate surface to the case when the major axis faces the Au electrode in neutral C_70_ at the point of relative stability, in the former case, the negative surface charge is more favorable for adsorption than the positive surface charge (Figure 4c), similar to its anion.

Regardless of the sign of the surface charge, with respect to electron transport, the major axis arrangements of C_70_ fullerene molecules facing the gate electrodes would be more favorable than the minor -axis arrangement, based on the delocalization of frontier orbitals of both C_70_ and its anion, as discussed later.

Our model calculations show that the stability of the C_70_ fullerene adsorption on the gate-induced polarity charge depends on the polarity and size of the surface-induced charges. It also suggests that the energy mode of 11 meV observed in [16] would be caused by the rotational transition of the C_70_ fullerene vertical flip isomers facing the gate electrode molecule between Au electrodes, implying that C_70_ underwent a change in orientation when a specific gate voltage was switched on in the SET experiment conducted.

**Figure 5:**
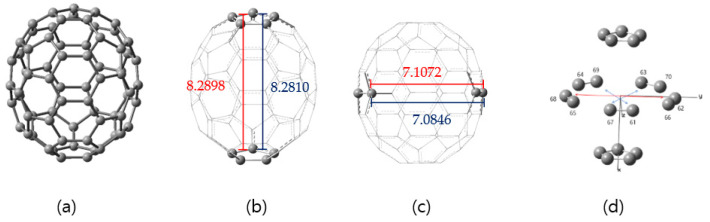
(**a**) The ball and stick structure of C_70_ fullerene skeleton. (**b**) Distances (Å) between two pentagons of the top and bottom poles and for the diameter of the minor axis on the equatorial zone are: (**b**) 8.281 and (**c**) 7.085 for neutral C_70_ and (**b**) 8.290 and (**c**) 7.107 for the C_70_ anion. The major axis and the minor axis are represented by x and y (or z), respectively. (**d**) The cartesian coordinates and the length of the y-axis on the equator for C_70_ fullerene skeleton were shown.

As shown in Figure 5, the change in interatomic distances in the atomic arrangement structures shows that for anions (i.e., when electron doping takes place) the structural deformation is increased in certain directions and decreases in other directions. This indicates that in the case of molecular devices with C_70_ inserted between the electrodes, the distance between the electrodes and the molecules could possibly be increased in a specific direction by electron doping.

In our calculations, by applying the bouncing-ball mode model of C_60_ [8], we obtain an energy mode of 4.7 meV for C_70_ with a force constant of 70 N/m using a harmonic potential model. Thus, in this energy mode, the current in C_70_ molecular devices could be explained as being analogous to the maximum current when a 5 meV gate voltage is applied to the C_60_ transistor [8]. Thus, it is suggested that the energy modes near 5 meV for the C_70_ molecular device [15] would be equivalent to the electron transport mechanism of C_60_ molecular devices [8].

### 3.3. Charge and Spin Density Sites and Frontier Orbitals of C_70_ Orientation Isomers and Anions

**Figure 6:**
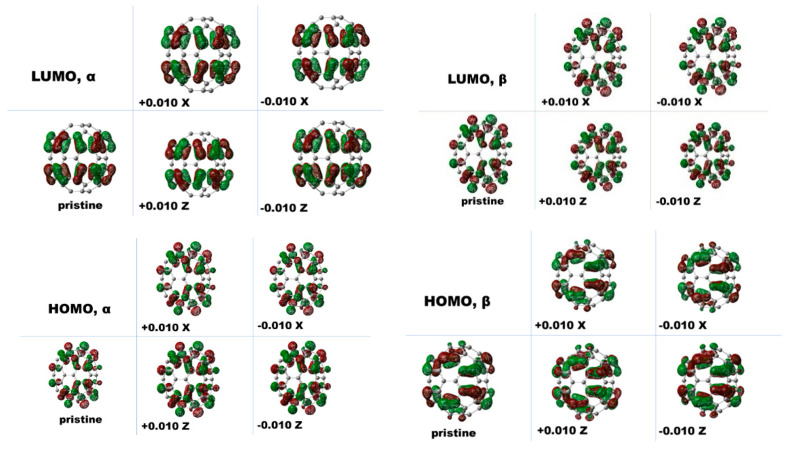
Frontier molecular orbitals diagram of the two orientation isomers of C_70_ fullerene anion systems. The pristine anion is calculated at the B3LYP/6-31G (d, p) level including GD3. The others are for the B3LYP/6-31G (d, p) level including GD3 plus the point charges model. Here, X and Z represent the major and minor axis directions facing the point charges, respectively. The distance between the model point charges (δ = 0.010) and the closest atoms of fullerene to the surface is 2.8 Å.

Two orientations of the C_70_ vertical flip isomers facing the gate electrode between the electrodes can form when the C_70_ cage interacts with the surface point charges in the two prolate spheroidal directions. In the case of both C_70_ and the C_70_ anion, under the surface point charge model, the total number of electrons of the two orientation isomer species to the point charge model is the same. Figure 6 shows the frontier orbitals of the C_70_ anion and its C_70_ vertical flip isomers facing the gate electrode between the electrodes using the point charge model. Because an electron orbital nodal plane exists between the lower pentagon and the upper pentagon in the longer major axis (*x*-axis) direction for the frontier orbitals, it would prevent the flow of electrons along this direction. By contrast, there are no such nodes in the major axis direction parallel to the equator and perpendicular to the x direction; therefore, there is little impediment to the flow of electrons in the major axis direction of C_70_ facing the gate electrode when considering frontier orbitals.

Although the peak near 11 meV in the histogram of C_70_ levels was not prominent, one 11 meV energy mode was observed in one of eight single C_70_ transistors in [16], unlike the C_140_ single-molecule device, in which the 11 meV mode was prevalent [16]. Thus, explaining this difference should be interesting. Our calculations suggest that the 11 meV energy mode in the C_70_ single-molecule device is due to the vertical flip C_70_ rearrangement from the major axis C_70_ isomer facing the Au electrodes to the major axis direction isomer facing the gate electrode. As previously demonstrated, the electron transport mechanism near the C_70_ 5 meV energy mode was similar to that of a C_60_ device. In addition, based on the node characteristics of the frontier orbitals, the findings of the nodal planes in the lowest unoccupied molecular orbitals (LUMOs) of C_70_ and both the highest occupied molecular orbitals (HOMOs) and the LUMO of the C_70_ anion suggest that the electron tunneling of pristine C_70_ prolate spheroidal fullerene and its anion may improve when the major axis orientation faces the gate electrode than when the major axis orientation faces the Au source and drain electrodes. Therefore, we could suggest that a single-C_70_ transistor would exhibit an energy barrier to the major axis orientation of the C_70_ isomers facing the gate electrode when flipped from the major axis orientation facing the Au electrodes, which would result in an energy mode of approximately 11 meV, as shown in Figure 4.

Density functional theory (DFT) calculations for the C_70_ fullerene indicate that iso-surfaces of the frontier orbitals of gas-phase C_70_ chemical species occur when the wave functions of both the HOMO and LUMO are mostly delocalized to the fullerene cage, except for the nodal plane at the equator region. The isosurfaces of frontier orbitals for C_70_ vertical flip isomers facing the point charges using the model of the gate electrode between the electrodes similarly show that each wave function of the HOMO and LUMO levels has the nodal plane of the equator region, indicating that an energy barrier exists for one orientation of the C_70_ vertical flip isomers facing the gate electrode, which could inhibit the electron channel needed to bridge the junction. In pristine C_70_, DFT predicts that LUMO-dominated transport would occur at the C_70_ vertical flip isomer facing the gate electrode on the major axis facing the point charges. We can qualitatively suggest that from the viewpoint of frontier orbitals, electron tunneling arises from the rotational vertical flip rearrangement of the orientation of the C_70_ fullerene, implying that the orientation phase is changed from the major axis orientation facing the Au electrodes to the major axis facing the gate electrode between the electrodes.

**Figure 7:**
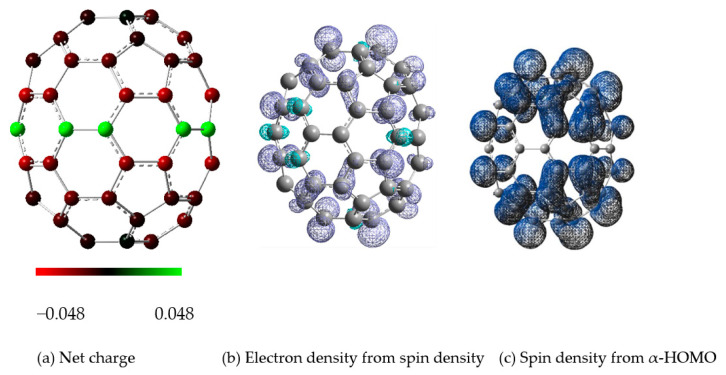
(**a**) Net charge of C_70_^−^, (**b**) electron density from spin density (= electron density from α-spin density minus electron density from β-spin density) of C_70_^−^, and (**c**) spin density from α-HOMO for C_70_^−^ showing qualitatively the unpaired electron distributions, in which C_70_^−^ is the optimized structure at the B3LYP/6-31G (d, p) level including the empirical dispersion interaction GD3.

Finally, we examined the distribution of the charge and electron spin density on the carbon atoms of the fullerene frame and the point charge model, which slightly affects the charge and spin density of C_70_. Thus, as shown in Figure 7, we show the distribution of the net charge of C_70_^−^, electron density from spin density (= electron density from α-spin density minus electron density from β-spin density) of C_70_^−^, and spin density from α-HOMO on the surface frame of the pristine C_70_^−^ anion. The numerical values of the charge and electron spin density for two C_70_ vertical flip isomers facing the gate electrode between the electrodes in a point charge model are almost the same, as shown in the Supplementary Material (See Table S2). Here, we should keep in mind that for heteronuclear molecules, the information given by Mulliken’s charges is hardly informative, because Mulliken population analysis computes charges by dividing orbital overlap evenly between the two atoms involved, implying that it is generally known to be highly dependent on the basis set [32,33].

In conclusion, first, our manuscript explained the 11 meV energy mode by using the rotation mode of C_70_ anion coupling the electric field of gate electrode, by using the total energy difference between different orientation conformers of C_70_ which depends on the coupling strength to the gate electrode. Secondly, the resonant conversion of electronic energy to C_70_ tuning with rotation (but without involving vibration) would be a likely process which was recently well known [34]. In addition, we should keep in mind that the relative stability of the orientation of C_70_ vertical flip isomers facing the gate electrode between the electrodes is different from the stability of a gas-phase C_70_ molecule with respect to energetics. This is in line with a study reporting that the combination of electronic and metal electrode effects significantly changes the stereochemical properties of relatively simple chemical species (atoms and molecules) in fullerenes [19].

In the future, we hope to apply our approach to real-world applications, such as those involving metal atoms and dielectric effects in a single-molecule device. We also would like to extend our simulations using skeletal rearrangements from the point of view of the orientation conformers to understand single electron tunneling of other types of molecules in a SET by considering the van der Waals interaction with the electrodes in more detail. However, it should be reminded that the 11 meV energy mode in reference [16] might be attempted to be interpreted as a coupling between the C_70_ and the terminal electrodes from a different perspective in the near future.

## Data Availability

Not applicable.

## Supplementary Materials

The following are available online at https://www.mdpi.com/article/10.3390/nano11112995/s1, Table S1: Atomic cartesian coordinates for optimized geometric structures of C70 fullerene and its anion using B3LYP/6-31G (d, p) including with the empirical dispersion GD3, Table S2: Numerical values of atomic charge and electron spin density for two C70 vertical flip isomers facing the point charges as a model of the gate electrode between the electrodes at the B3LYP/6-31G (d, p) level including the empirical dispersion interaction GD3 at the distance (2.8Å) between the model surface of point charges δ and the closest atoms of C_70_ chemical species to the point charges.
