# Supplementary material for: Electromechanical Characteristics by a Vertical Flip of C70 Fullerene Prolate Spheroid in a Single-Electron Transistor: Hybrid Density Functional Methods
Source: Nanomaterials (Basel). 2021 Nov 8;11(11):2995. doi: 10.3390/nano11112995 (PMC8623830; doi:10.3390/nano11112995)
Supplement: Supplementary file 1 [file nanomaterials-11-02995-s001.zip › nanomaterials-1415554-supplementary.pdf]

nanomaterials

Supplementary Materials

Electromechanical Characteristics by a Vertical Flip of C_{70} Fullerene Prolate Spheroid in a Single-Electron Transistor: Hybrid Density Functional Methods

Jong Woan Choi [1], Changhoon Lee [2,*], Eiji Osawa [3], Ji Young Lee [4,*], Jung Chul Sur [1,*], and Kee Hag Lee [4,*]

1 Department of Semiconductor and Display, Wonkwang University, Iksan 54538, Korea; jangja21@wku.ac.kr
2 Max Planck POSTECH Center for Complex Phase of Materials, Pohang University of Science and Technology, Pohang 37673, Korea
3 Nanocarbon Research Institute, Shinshu University, Ueda, Nagano 386-8567, Japan; osawa@nano-carbon.jp
4 Department of Chemistry, Nanoscale Sciences and Technology Institute, Nanocarbon R&D Institute, Wonkwang University, Iksan 54538, Jeonbuk, Korea
* Correspondence: chlee0887@postech.ac.kr (C.L.); ljy@wku.ac.kr (J.Y.L.); jcsur@wku.ac.kr (J.C.S.); khlee@wku.ac.kr (K.H.L.)

Table S1. Atomic cartesian coordinates for optimized geometric structures of C_{70} fullerene and its anion using B3LYP/6-31G (d, p) including with the empirical dispersion GD3.

C_{70}						C_{70}^{-} anion		
Atomic labelling number	x		y	z		x	y	z
1	-3.9703		0.7260	0.9993		-3.9817	-1.0052	0.7194
2	-3.9704		1.1748	-0.3818		-3.9704	0.3849	1.1655
3	-3.9704		0.0000	-1.2353		-3.9901	1.2419	-0.0001
4	-3.9704		-1.1748	-0.3818		-3.9705	0.3847	-1.1655
5	-3.9703		-0.7260	0.9993		-3.9817	-1.0053	-0.7193
6	3.9703		0.7260	0.9993		3.9817	-1.0052	0.7194
7	3.9703		-0.7260	0.9993		3.9817	-1.0053	-0.7192
8	3.9704		-1.1748	-0.3818		3.9705	0.3847	-1.1655
9	3.9704		0.0000	-1.2353		3.9901	1.2419	-0.0001
10	3.9704		1.1748	-0.3818		3.9704	0.3849	1.1655
11	-3.2225		1.4194	1.9536		-3.2254	-1.9615	1.4214
12	-3.2226		2.2968	-0.7463		-3.2182	0.7456	2.2880
13	-3.2225		0.0000	-2.4148		-3.2301	2.4323	-0.0001
14	-3.2226		-2.2968	-0.7463		-3.2182	0.7454	-2.2881
15	-3.2225		-1.4194	1.9536		-3.2254	-1.9617	-1.4212
16	3.2225		1.4194	1.9536		3.2254	-1.9615	1.4214
17	3.2225		-1.4194	1.9536		3.2254	-1.9617	-1.4212
18	3.2226		-2.2968	-0.7463		3.2182	0.7454	-2.2881

19	3.2225		0.0000	-2.4148	3.2301	2.4323	-0.0001
20	3.2226		2.2968	-0.7463	3.2182	0.7456	2.2880
21	-2.4431		0.6943	2.9361	-2.4462	-2.9417	0.6948
22	-2.4431		3.0069	0.2469	-2.4410	-0.2464	2.9997
23	-2.4430		1.1641	-2.7833	-2.4510	2.7944	1.1580
24	-2.4432		-2.2876	-1.9672	-2.4400	1.9699	-2.2801
25	-2.4431		-2.5777	1.5676	-2.4479	-1.5766	-2.5727
26	2.4431		2.5777	1.5676	2.4479	-1.5763	2.5728
27	2.4431		-0.6943	2.9361	2.4462	-2.9417	-0.6946
28	2.4431		-3.0069	0.2469	2.4410	-0.2467	-2.9997
29	2.4430		-1.1641	-2.7833	2.4510	2.7943	-1.1583
30	2.4432		2.2876	-1.9672	2.4400	1.9701	2.2799
31	2.4431		0.6943	2.9361	2.4462	-2.9417	0.6948
32	2.4431		3.0069	0.2469	2.4410	-0.2464	2.9997
33	2.4430		1.1641	-2.7833	2.4510	2.7944	1.1580
34	2.4432		-2.2876	-1.9672	2.4400	1.9699	-2.2801
35	2.4431		-2.5777	1.5676	2.4479	-1.5766	-2.5727
36	-2.4431		2.5777	1.5676	-2.4479	-1.5763	2.5728
37	-2.4431		-0.6943	2.9361	-2.4462	-2.9417	-0.6946
38	-2.4431		-3.0069	0.2469	-2.4410	-0.2467	-2.9997
39	-2.4430		-1.1641	-2.7833	-2.4510	2.7943	-1.1583
40	-2.4432		2.2876	-1.9672	-2.4400	1.9701	2.2799
41	-1.2064		1.4143	3.1669	-1.2079	-3.1836	1.4139
42	-1.2064		3.4489	-0.3665	-1.2087	0.3654	3.4384
43	-1.2063		0.7172	-3.3931	-1.2069	3.4056	0.7155
44	-1.2064		-3.0058	-1.7308	-1.2090	1.7398	-3.0018
45	-1.2064		-2.5746	2.3236	-1.2075	-2.3276	-2.5659
46	1.2064		2.5746	2.3236	1.2075	-2.3273	2.5662
47	1.2064		-1.4143	3.1669	1.2079	-3.1837	-1.4135
48	1.2064		-3.4489	-0.3665	1.2087	0.3651	-3.4384
49	1.2063		-0.7172	-3.3931	1.2069	3.4056	-0.7159
50	1.2064		3.0058	-1.7308	1.2090	1.7401	3.0017
51	1.2064		1.4143	3.1669	1.2079	-3.1836	1.4139
52	1.2064		3.4489	-0.3665	1.2087	0.3654	3.4384
53	1.2063		0.7172	-3.3931	1.2069	3.4056	0.7155
54	1.2064		-3.0058	-1.7308	1.2090	1.7398	-3.0018
55	1.2064		-2.5746	2.3236	1.2075	-2.3276	-2.5659
56	-1.2064		2.5746	2.3236	-1.2075	-2.3273	2.5662
57	-1.2064		-1.4143	3.1669	-1.2079	-3.1837	-1.4135
58	-1.2064		-3.4489	-0.3665	-1.2087	0.3651	-3.4384

59	-1.2063		-0.7172	-3.3931	-1.2069	3.4056	-0.7159
60	-1.2064		3.0058	-1.7308	-1.2090	1.7401	3.0017
61	0.0000		0.7352	3.4859	0.0000	-3.5002	0.7361
62	0.0000		3.5420	0.3778	0.0000	-0.3770	3.5276
63	0.0000		1.4540	-3.2520	0.0000	3.2618	1.4520
64	0.0000		-2.6437	-2.3879	0.0000	2.3927	-2.6399
65	0.0000		-3.0874	1.7761	0.0000	-1.7771	-3.0756
66	0.0000		3.0874	1.7761	0.0000	-1.7768	3.0758
67	0.0000		-0.7352	3.4859	0.0000	-3.5003	-0.7357
68	0.0000		-3.5420	0.3778	0.0000	-0.3774	-3.5276
69	0.0000		-1.4540	-3.2520	0.0000	3.2617	-1.4523
70	0.0000		2.6437	-2.3879	0.0000	2.3929	2.6396

Table S2. Numerical values of atomic charge and electron spin density for two C_{70} vertical flip isomers facing the point charges as a model of the gate electrode between the electrodes at the B3LYP/6-31G (d, p) level including the empirical dispersion interaction GD3 at the distance (2.8Å) between the model surface of point charges δ and the closest atoms of C_{70} chemical species to the point charges.

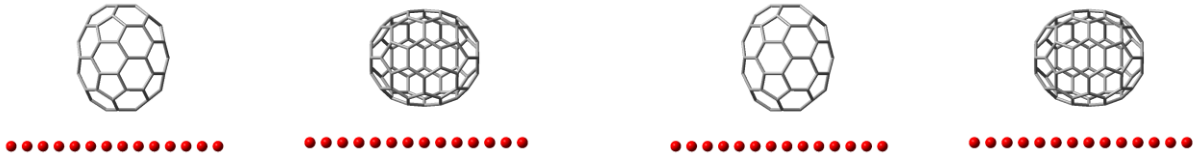

Point charges	δ = +0.01				δ = -0.01			
Number	Charges	spin densities	Charges	spin densities	Charges	spin densities	Charges	spin densities
1	-0.024672	0.059747	-0.013999	0.054862	-0.008236	0.055633	-0.018668	0.058278
2	-0.016361	-0.016169	-0.007734	-0.013536	-0.002925	-0.015604	-0.011589	-0.017958
3	-0.031489	0.110164	-0.019763	0.104449	-0.011883	0.102544	-0.023207	0.104686
4	-0.016324	-0.016126	-0.009454	-0.01823	-0.002963	-0.01563	-0.009839	-0.013718
5	-0.024615	0.059668	-0.015701	0.058595	-0.00829	0.055686	-0.01698	0.054804
6	-0.013331	0.05495	-0.01429	0.054994	-0.019555	0.060497	-0.018375	0.058121
7	-0.013323	0.054926	-0.01632	0.059001	-0.019563	0.060516	-0.016365	0.054469
8	-0.007404	-0.015501	-0.010017	-0.018245	-0.011872	-0.016266	-0.009275	-0.013707
9	-0.018027	0.101391	-0.020193	0.104861	-0.025305	0.111369	-0.02278	0.104296
10	-0.007408	-0.015545	-0.007885	-0.013556	-0.011869	-0.016237	-0.011438	-0.017934
11	-0.022709	0.015908	-0.015916	0.01342	-0.013396	0.007763	-0.020023	0.008999
12	-0.020996	0.002832	-0.014062	0.004326	-0.012045	0.004044	-0.018987	0.002543
13	-0.024367	0.023925	-0.017484	0.014845	-0.013705	0.00975	-0.020274	0.0164

14	-0.020943	0.002854	-0.018815	0.002755	-0.012101	0.004028	-0.014211	0.004146
15	-0.022576	0.015975	-0.017819	0.007737	-0.013531	0.007714	-0.018084	0.01441
16	-0.015242	0.008854	-0.016121	0.013834	-0.02085	0.01464	-0.019826	0.008645
17	-0.015235	0.008887	-0.01847	0.007965	-0.020858	0.014605	-0.017427	0.01412
18	-0.013978	0.003938	-0.019546	0.002664	-0.019064	0.002942	-0.013478	0.004206
19	-0.01611	0.011373	-0.017759	0.015403	-0.021929	0.021992	-0.02	0.015845
20	-0.013985	0.003926	-0.014163	0.004274	-0.019054	0.002957	-0.01889	0.002613
21	-0.018007	-0.011259	-0.014968	-0.005809	-0.015073	-0.008837	-0.018348	-0.012512
22	-0.022496	0.024613	-0.016858	0.012732	-0.018459	0.015952	-0.023997	0.027207
23	-0.021553	0.01738	-0.016315	0.010431	-0.017099	0.020847	-0.022475	0.028536
24	-0.019371	-0.007169	-0.020121	-0.008508	-0.015166	-0.012377	-0.014449	-0.010957
25	-0.0237	0.039362	-0.027141	0.049951	-0.020101	0.036092	-0.016781	0.026511
26	-0.019296	0.035845	-0.018082	0.026395	-0.024522	0.039735	-0.025675	0.048547
27	-0.014517	-0.009026	-0.015797	-0.012584	-0.018567	-0.011089	-0.017468	-0.006331
28	-0.01784	0.01627	-0.027396	0.026426	-0.023119	0.024245	-0.013547	0.013775
29	-0.017188	0.020013	-0.02063	0.030025	-0.021474	0.018302	-0.018302	0.010492
30	-0.015085	-0.011722	-0.014594	-0.011512	-0.019445	-0.007959	-0.019978	-0.007619
31	-0.014522	-0.009085	-0.015105	-0.005973	-0.018559	-0.011054	-0.018204	-0.012435
32	-0.017878	0.01638	-0.016953	0.013067	-0.02308	0.024129	-0.023891	0.026764
33	-0.017208	0.02018	-0.016395	0.010294	-0.021457	0.018167	-0.022392	0.028672
34	-0.01507	-0.011742	-0.020493	-0.008239	-0.019464	-0.007925	-0.014092	-0.011109
35	-0.019248	0.035673	-0.027786	0.050263	-0.024571	0.039885	-0.016158	0.026357
36	-0.023894	0.039562	-0.017991	0.0263	-0.019913	0.035949	-0.025777	0.048726
37	-0.017939	-0.011223	-0.015534	-0.01254	-0.015142	-0.008878	-0.017725	-0.006461
38	-0.02239	0.024468	-0.026793	0.025928	-0.018561	0.016056	-0.014125	0.014111
39	-0.021526	0.017202	-0.020387	0.030092	-0.017121	0.020988	-0.01854	0.010388
40	-0.019401	-0.007126	-0.014519	-0.011664	-0.015138	-0.012391	-0.020064	-0.00735
41	-0.040246	0.056933	-0.03685	0.037774	-0.04107	0.052381	-0.044293	0.070168
42	-0.035889	0.0047	-0.033562	-0.000747	-0.037461	0.005707	-0.039846	0.012285
43	-0.036993	0.010802	-0.036741	0.023934	-0.037258	0.008299	-0.037685	-0.002828
44	-0.039737	0.05336	-0.047426	0.06745	-0.040596	0.051807	-0.033004	0.038853
45	-0.033759	-0.021579	-0.037456	-0.025498	-0.035309	-0.020948	-0.031718	-0.015543
46	-0.033166	-0.020858	-0.032915	-0.015126	-0.035897	-0.021712	-0.036363	-0.024918
47	-0.038572	0.052234	-0.042641	0.07135	-0.042748	0.057076	-0.038721	0.038704
48	-0.035172	0.005736	-0.045089	0.012808	-0.038179	0.004686	-0.028448	-0.000452
49	-0.035589	0.008342	-0.035427	-0.003524	-0.038663	0.010801	-0.038794	0.022646
50	-0.038394	0.051325	-0.036121	0.038277	-0.041951	0.053938	-0.044073	0.066034
51	-0.03864	0.052504	-0.036925	0.038001	-0.042683	0.056858	-0.044217	0.069912
52	-0.035215	0.005848	-0.033571	-0.000775	-0.038141	0.004604	-0.03984	0.012329
53	-0.035572	0.008096	-0.036782	0.024068	-0.03868	0.010999	-0.037651	-0.00291

54	-0.038351	0.051074	-0.047573	0.067523	-0.041991	0.054148	-0.032856	0.038779
55	-0.033122	-0.020783	-0.037608	-0.025584	-0.035942	-0.021773	-0.031557	-0.01553
56	-0.033867	-0.021665	-0.032903	-0.015116	-0.035198	-0.020894	-0.036369	-0.02498
57	-0.040126	0.056668	-0.042478	0.071129	-0.041186	0.052586	-0.038881	0.03889
58	-0.035827	0.004597	-0.044948	0.012888	-0.037522	0.005789	-0.028597	-0.000494
59	-0.03701	0.011058	-0.035358	-0.003621	-0.037244	0.008112	-0.038868	0.022796
60	-0.039787	0.053606	-0.036092	0.038176	-0.04055	0.051603	-0.044102	0.066142
61	0.048648	-0.022349	0.048041	-0.014624	0.046674	-0.0223	0.047165	-0.029572
62	0.0458	-0.007994	0.046563	-0.004467	0.043795	-0.007926	0.043052	-0.011848
63	0.048125	-0.003321	0.049821	-0.009912	0.046419	-0.003353	0.044743	0.002377
64	0.04681	-0.02533	0.044687	-0.032873	0.045293	-0.025694	0.047422	-0.018848
65	0.046752	0.00572	0.03956	0.007616	0.044463	0.005841	0.051655	0.003481
66	0.046684	0.005752	0.04809	0.003385	0.044533	0.00582	0.043196	0.007302
67	0.048647	-0.022223	0.049445	-0.030139	0.046677	-0.022396	0.04584	-0.015008
68	0.045843	-0.007932	0.038944	-0.012131	0.04375	-0.007972	0.05069	-0.004639
69	0.048142	-0.003439	0.04559	0.002794	0.046401	-0.003266	0.048885	-0.009421
70	0.046811	-0.025456	0.047093	-0.01853	0.045292	-0.025594	0.044936	-0.032279
